# A T7 autogene-based hybrid mRNA/DNA system for long-term shRNA expression in cytoplasm without inefficient nuclear entry

**DOI:** 10.1038/s41598-019-39407-8

**Published:** 2019-02-28

**Authors:** Seo Young Kwak, Hee Dong Han, Hyung Jun Ahn

**Affiliations:** 1 Center for Theragnosis, Biomedical Research Institute, Korea Institute of Science and Technology, Seoul, South Korea; 2 Department of Immunology, School of Medicine, Konkuk University, Chungju, South Korea

## Abstract

The transient silencing effects currently demonstrated by nonviral siRNA delivery systems limit the therapeutic utility of RNAi, but it remains a technical challenge to prolong duration of gene silencing. We have developed a T7 autogene-based hybrid mRNA/DNA system to enable long-term expression of shRNA in cytoplasm *in vitro* and *in vivo*. This hybrid mRNA/DNA system consists of T7 polymerase (T7pol) mRNA, pT7/shRNA-encoding DNA fragment and T7 autogene plasmid, and it can generate higher levels of T7pol proteins, compared to pCMV-triggering T7 autogene system, especially without the need of nuclear entry of any gene. A large amount of T7pol proteins produced are used to induce pT7-driven expression of shRNA in cytoplasm, and through cellular processing of RNA hairpins, mature siRNAs are generated for more than 13 days. We here demonstrate that a single liposomal delivery of this hybrid system leads to the long-term silencing effects *in vitro* and *in vivo*, in contrast to the conventional siRNA methods relying on the repeated administrations every 2 or 3 days. These sustainable shRNA expression properties in cytoplasm can provide an efficient strategy to address the limitations caused by shRNA-encoding plasmid DNA systems such as low nuclear entry efficiency and short-term silencing effect. The development of long-term shRNA expression system *in vivo* could scale down administration frequency of RNAi therapeutics in the treatment of chronic diseases, thereby increasing its clinical utility.

## Introduction

RNA interference (RNAi) has recently emerged as a promising therapeutic tool to suppress the specific genes in the therapeutic utilities and gene functional studies^1^. Since the chemically synthesized siRNAs with short length of oligonucleotides (21- to 23-bp) were used to trigger RNAi in the eukaryotic cells, a variety of siRNA delivery systems have been devised to induce RNAi, including nonviral or viral vectors such as nanoparticles^2^, liposomes^3^, and viruses^4^. However, it has remained great challenge to induce the long-term silencing on target gene by using siRNA-mediated RNAi. Usually, downregulation of gene expression induced by siRNA lasts for only 2–4 days in cell culture system^5^. Thus, clinical applications of siRNA have been limited only to the transient suppression effects on the disease-related gene expression, although numerous attempts that use exogenous siRNAs as the potential therapeutic agents for patients have received great attention.

As an alternative approach for the long-term silencing effects, a variety of recombinant viral vectors including adenoviral and lentiviral vectors have been proven to be efficient in realizing gene silencing for extended periods *in vitro* and *in vivo*^6^. For example, adenoviral vectors encoding shRNA against PTTG1 gene significantly inhibited the growth of carcinoma cells *in vivo* in the long term^7^. However, safety concerns such as unwanted immune responses and insertion mutagenesis as well as difficulties of large-scale manufacturing have limited the use of viral vectors for siRNA delivery in clinical applications^8,9^.

Ever since it has been reported that siRNAs could be expressed as short hairpin RNA (shRNA) from RNA polymerase III (pol III) promoters cloned into plasmids^4,10^, many researchers have devised the endogenous nonviral expression systems of shRNA in cells, and concentrated on development of plasmid DNAs for promoter-based expression of shRNA in nucleus^11^. Although there are several types of pol III promoters that affect the sequences and structures of RNA hairpins, U6 and H1 promoters, derived from U6 small nuclear RNA (snRNA) and H1 RNA genes, have been widely used for shRNA expression due to their simple and strong transcriptional activity^12,13^. However, a major obstacle restricting pol III-mediated expression of shRNA is the inefficient transfer of exogenous plasmid DNAs from the cytoplasm to the nucleus with only 1% of plasmid DNAs reaching the nucleus after cytoplasmic internalization^14^. A number of cells are post-mitotic or non-dividing, and nuclear transport efficiency is significantly lower in these cells because of the limited breakdown of nuclear envelope^15^. This is especially true for the *in vivo* applications. Therefore, the levels of shRNA expression mediated by nonviral vectors are significantly lower than those by viral vectors^16^. Furthermore, the shRNA-expressing DNA plasmid is not superior in its ability to sustain shRNA expression.

As one strategy for improving these disadvantages found in the nuclear expression systems, a cytoplasmic shRNA expression system has been devised, which can transcribe shRNA in the cytoplasm, not in the nucleus. For example, pCMV_T7pol/pT7_shRNA plasmid DNAs, where the transcription of shRNA under the control of T7 promoter depends on the T7pol driven by cytomegalovirus (CMV) promoter, have been reported to mediate cytoplasmic expression of shRNA and thus suppress the exogenous reporter gene expression *in vitro*^17^. However, these works also require nuclear entry of plasmid DNAs to induce the expression of T7pol, which compromises the usefulness of this system^15,18^. Moreover, there has been no report on the improvement in the duration of shRNA expression.

Since bacteriophage T7pol has the transcriptional activity in cytoplasm without the requirement of cellular cofactors, this property has been utilized in the T7-based autogene systems to mediate high levels of transgene expression under the control of T7 promoter^19^. After initial triggering of T7 autogene by T7pol proteins, a large amount of T7pol proteins can be produced in an autocatalytic positive feedback loop, finally driving high levels of expression of a co-delivered transgene under the control of T7 promoter. For example, T7-based autogenes were used to induce the cytoplasmic expression of chloramphenicol acetyltransferase (CAT), and high level of CAT expression could be observed up to 5 days after transfection^20^. Despite many successful examples of T7 autogene systems utilized for high expression of transgene in cytoplasm, their application for cytoplasmic shRNA expression has not been reported yet.

Here, we have developed and evaluated a T7 autogene-based hybrid mRNA/DNA system, where pT7-driven cytoplasmic expression of shRNA can be induced by self-replenished T7pol proteins. This hybrid system, consisting of T7 polymerase (T7pol) mRNA, pT7/shRNA-encoding DNA fragment and T7 autogene plasmid, enables higher levels of T7pol expression than pCMV-triggering T7 autogene system, especially without nuclear entry of any genes. A large quantity of T7pol proteins produced can induce the cytoplasmic expression of shRNA for a prolonged period of time, and ultimately, mature siRNAs, the processed form of shRNAs, are produced for more than 13 days through the function of cellular RNAi machinery. Furthermore, systemic administration of liposomes containing this hybrid system into tumor-bearing mice shows the long-term silencing effects at the tumor sites, especially without the repeated administration.

For quantitative evaluation of long-term silencing effects *in vitro* and *in vivo*, red fluorescence protein (RFP) expressing B16F10 (B16F10/RFP) melanoma cells were selected, and the suppression of RFP gene expression was examined by using quantitative measurement of residual RFP mRNA or protein over time. Also, a flow cytometry and noninvasive optical imaging technique was employed to analyze the effects of RFP gene silencing in the tumor cells or tissues. On the B16F10/RFP tumor-bearing mice, we investigated whether the intravenously administered hybrid system could efficiently downregulate RFP gene at tumor sites without the periodically repeated injections, while comparing with those of conventional siRNA methods relying on the frequent administration. Finally, we investigated the activation of innate immunogenicity of auto_shRNA system on the human PBMCs *in vitro* and on the C57BL/6J mice, respectively.

## Results

### Design and synthesis of T7 autogene-based hybrid system

**Figure 1:**
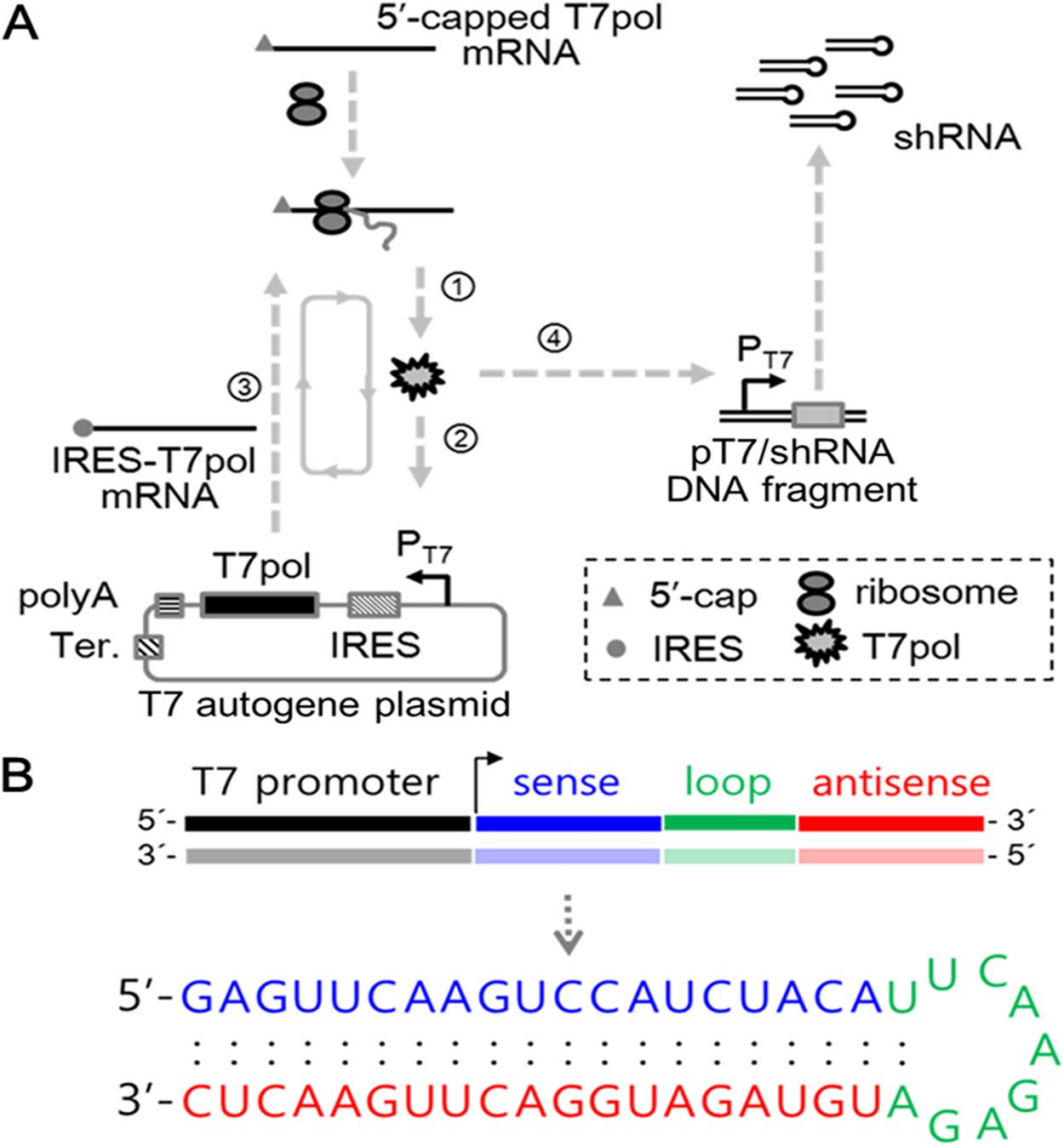
Schematic diagram of T7 autogene-based hybrid mRNA/DNA system and structure of produced shRNA. (**A**) The mechanism of hybrid mRNA/DNA system in the transfected cells is illustrated. 5′-capped T7pol mRNAs are (1) translated into T7pol proteins *via* cellular translational machinery, and then initial binding (2) of T7pol proteins to T7 promoter on T7 autogene plasmid transcribes IRES-fused T7pol mRNA (3), which is subsequently (1) translated into T7pol protein *via* cellular translational machinery. After T7pol proteins trigger the autocatalytic positive feedback loop, their iterative binding to T7 promoters on T7 autogene plasmids results in the large quantities of T7pol proteins. A portion of T7pol proteins exiting the autocatalytic loop bind to T7 promoters of pT7/shRNA DNA fragments, and induce the expression of shRNA in the cytoplasm. (**B**) The predicted hairpin structure of RNA expressed from pT7/shRNA DNA fragment. The RNA sequences of hairpin directed against red fluorescence protein (RFP) mRNA are depicted: 19-nt sequences (blue) from the target mRNA are separated by a short loop (green) from the reverse complement (red) of the same sequences. The mfold web server was used for simulation of RNA folding (http://mfold.rna.albany.edu/?q=mfold/RNA-Folding-Form).

For self-amplifying regeneration of T7pol proteins, we constructed T7 autogene plasmid, in which T7pol-encoding gene is located downstream of T7 promoter and EMCV (Encephalomyocarditis virus) IRES (Internal Ribosomal Entry Site) elements, as well as upstream of polyA sequence and T7 terminator (Fig. 1A). Since mRNA produced in the cytoplasm lacks a 5′-cap that stabilizes transcripts and recruits the translational machinery^21^, the sequences of viral IRES elements, as an alternative regulatory moiety of 5′-cap on nuclear transcripts, were contained to enhance the recruitment of translational machinery, as described in the previous autogene system^22^. Next, we enzymatically synthesized 5′-capped T7pol mRNAs by incorporating anti-reverse cap analog (ARCA) into the *in vitro* transcription reaction. In the T7 autogene system, these T7pol mRNAs are responsible for triggering early expression of T7pol in the T7 autogene without any nuclear entry. Finally, we synthesized 64-bp of pT7-driven shRNA-encoding DNA fragment (pT7/shRNA DNA fragment) using an assembly PCR method. This DNA fragment was designed to specify 19-nt of sequences directed against RFP target mRNA, separated by a 9-nt of short loop from the reverse complementary of the same sequence.

The schematic diagram in Fig. 1A illustrates the mechanism of T7 autogene-based hybrid mRNA/DNA (*i.e*. auto_shRNA) system that can induce expression of shRNA from pT7/shRNA DNA fragment in the cytoplasmic compartment. Without the requirement of nuclear entry of any gene, the first round translation of T7pol is initiated from co-delivered T7pol mRNA *via* cellular translation machinery including ribosome. The initial binding of T7pol protein to its cognate T7 promoter on T7 autogene plasmid generates IRES-fused T7pol transcript, which is then translated into T7pol protein. While repeating the autocatalytic positive feedback loop, the iterative binding of T7pol protein to T7 promoters on T7 autogene plasmids can produce the large quantities of T7pol proteins. Ultimately, these T7pol proteins are used to induce expression of shRNA by binding to T7 promoters on pT7/shRNA DNA fragment. According to the RNA mfold program available for the simulation of RNA folding, the resulting shRNA is predicted to fold back on itself to form a stem-loop structure (Fig. 1B).

### Physicochemical characteristics of liposomes loaded with hybrid mRNA/DNA system

**Figure 2:**
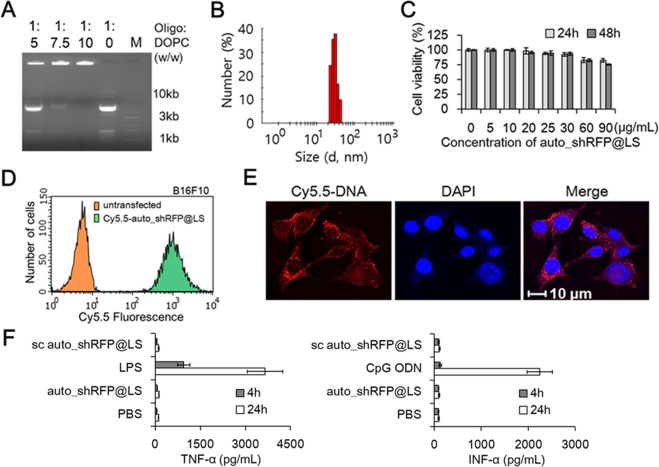
Biophysicochemical characterization of auto_shRNA@LS lipoplexes. (**A**) The electrophoretic mobility shift assay of oligonucleotides/DOPC lipoplexes at the specified weight ratios. While the molar ratio of each oligonucleotides was fixed (pT7/shRNA DNA fragment:T7pol mRNA:T7 autogene plasmid DNA = 0.4:1:1), a fixed amount of oligonucleotides were mixed with the various amounts of DOPC lipids (oligonucleotides:DOPC = 1:0, 1:5, 1:7.5, 1:10, w/w). At a weight ratio of 1:10, most oligonucleotides/DOPC complexes stayed stuck on the well of 1.5% agarose gels, but partially complexed or uncomplexed oligonucleotides at the lowered weight ratio moved down. These results indicate that total oligonucleotides completely complexed with DOPC lipids at a weight ratio of 1:10. Oligo and M represents oligonucleotides and DNA molecular markers, respectively. (**B**) Size distribution profile of auto_shRFP@LS lipoplexes measured on dynamic light scattering. (**C**) *In vitro* cytotoxicity studies of B16F10/RFP cells transfected with auto_shRFP@LS were examined 24 h and 48 h post-transfection using an MTT assay. Data represent the mean ± s.d. (n = 5). (**D**) Flow cytometry analysis of B16F10 cells transfected with Cy5.5-labeled auto_shRFP@LS lipoplexes. The percentage of B16F10 cells sorted within the Cy5.5 fluorescence gate region is about 100%. (**E**) Intracellular uptake studies of Cy5.5-labeled auto_shRFP@LS lipoplexes using confocal microscopic images on the B16F10 cells. Blue and red colors represent DAPI dyes and Cy5.5-labeled auto_shRFP@LS lipoplexes, respectively. (**F**) Proinflammatory cytokine activation of auto_shRFP@LS in human PBMCs. TNF-α and INF-α release was analyzed 4 h and 24 h after treatment with auto_shRFP@LS, PBS, scrambled (sc) auto_shRFP@LS, lipopolysaccharide, or CpG oligodeoxynucleotide. Data represent the mean ± s.d. (n = 5).

When considering systemic delivery of our auto_shRNA system to specific cells/tissues, the selection of an efficient delivery vehicle is essentially important due to its potential, undesirable side effects upon administration^23^. Usually, cationic liposomes can readily complex with the negative-charged oligonucleotides, but have several limitations unfavorable for their efficient delivery, probably caused by high affinity for endothelial cells, stable intracellular properties, or failure to release of oligonucleotide loads^24^. Herein, we selected the neutral DOPC liposomal vehicle, which has shown a successful systemic delivery capability for the loaded oligonucleotides in xenograft mouse models^25^. When a fixed amount of mRNA/DNA components of RFP-specific auto_shRNA (auto_shRFP) system (pT7/shRNA DNA fragment:T7pol mRNA:T7 autogene plasmid = 0.4:1:1, molar ratio) were complexed with DOPC liposomes at the various complexing ratios (oligonucleotides:DOPC = 1:0, 1:5, 1:7.5, 1:10, w/w), the electrophoretic mobility on gel retardation assay showed the formation of partially or fully complexed mRNA/DNA/liposome lipoplexes (*i.e*., auto_shRFP@LS) (Fig. 2A). At a weight ratio of 1:10, most mRNA/DNA oligonucleotides stayed stuck in the well without running down, indicating that all mRNA/DNA components are completely complexed with liposomes. Therefore, such a complexing ratio (oligonucleotides:liposomes = 1:10, w/w) was chosen as the optimal delivery formulation in the current studies.

Dynamic light scattering analysis revealed that auto_shRFP@LS lipoplexes had the nano-sized hydrodynamic diameter with 31.4 ± 1.2 nm, while their zeta potential was 1.1 mV (Fig. 2B). Also, MTT assay showed that there was no appreciable cytotoxicity in the lipoplexes-transfected B16F10/RFP cells both 24 h and 48 h post-transfection when monitored up to high concentration of lipoplexes such as 60 μg/mL (Fig. 2C). These results indicate that lipoplexes can be transfected to the cell culture systems in concentration ranges to induce the gene silencing without undesirable cytotoxicity.

Next, the cellular internalization of Cy5.5-labeled auto_shRFP@LS, prepared by loading the Cy5.5-dUTP-incorporated pT7/shRFP DNA fragments with other oligonucleotide components into liposomes, was investigated in the B16F10 cancer cells. On the flow cytometry analysis, most cells showed the Cy5.5 fluorescent signals 1 h post-transfection, indicating that lipoplexes substantially entered the cells (Fig. 2D). According to confocal microscopic images, it was clear that the internalized lipoplexes were located in the cytoplasmic compartment, but not in the nuclear compartment (Fig. 2E). When the naked Cy5.5-labeled pT7/shRNA DNA fragments were employed to track their cellular internalization, we could not find any Cy5.5 fluorescent signal within the cells (data not shown). Thus, these cellular uptake data indicate that auto_shRNA system can be efficiently transfected into cancer cells through DOPC liposomal vehicles.

### Innate immunogenicity studies of auto_shRNA@LS lipoplexes

In mammalian cells, the delivery of exogenous siRNA or siRNA/carriers can potentially stimulate the innate immune system, of which the activation depends on siRNA sequence and structure, delivery carrier, and cell type^26^. When administered into mammals or primary blood cell cultures, the exogenous siRNA/carriers may induce the release of inflammatory cytokines, which is highly associated with activation of innate immunogenicity, and consequently leads to undesirable side effects and considerable toxicities in humans. Thus, we examined the innate immunogenicity of auto_shRFP@LS lipoplexes on human PBMCs using an ELISA assay method. When we monitored the release of TNF-α on human PBMCs, lipopolysaccharides (LPS), a positive stimulus for activation of TNF-α, significantly elevated TNF-α levels both 4 h and 24 h post-transfection, whereas either auto_shRFP@LS or scrambled auto_shRFP@LS did not stimulate TNF-α induction above the basal levels (Fig. 2F). Similarly, either auto_shRFP@LS or scrambled auto_shRFP@LS did not cause any detectable INF-α induction both 4 h and 24 h post-transfection, while CpG oligodeoxynucleotides (ODN), a positive stimulus for INF-α induction, revealed a marked increase of INF-α. Since the time points are important for a precise evaluation of cytokine release, we also examined whether TNF-α or INF-α was released at the earlier time such as 1 h and 2 h post-transfection, but did not observe any appreciable release of either TNF-α or INF-α (data not shown). Therefore, these data suggest that auto_shRNA@LS lipoplexes have great potential to be developed as RNAi therapeutics due to the lack of innate immunogenicity.

### Higher and more sustained levels of T7pol by co-delivery of T7pol mRNA and T7 autogene

**Figure 3:**
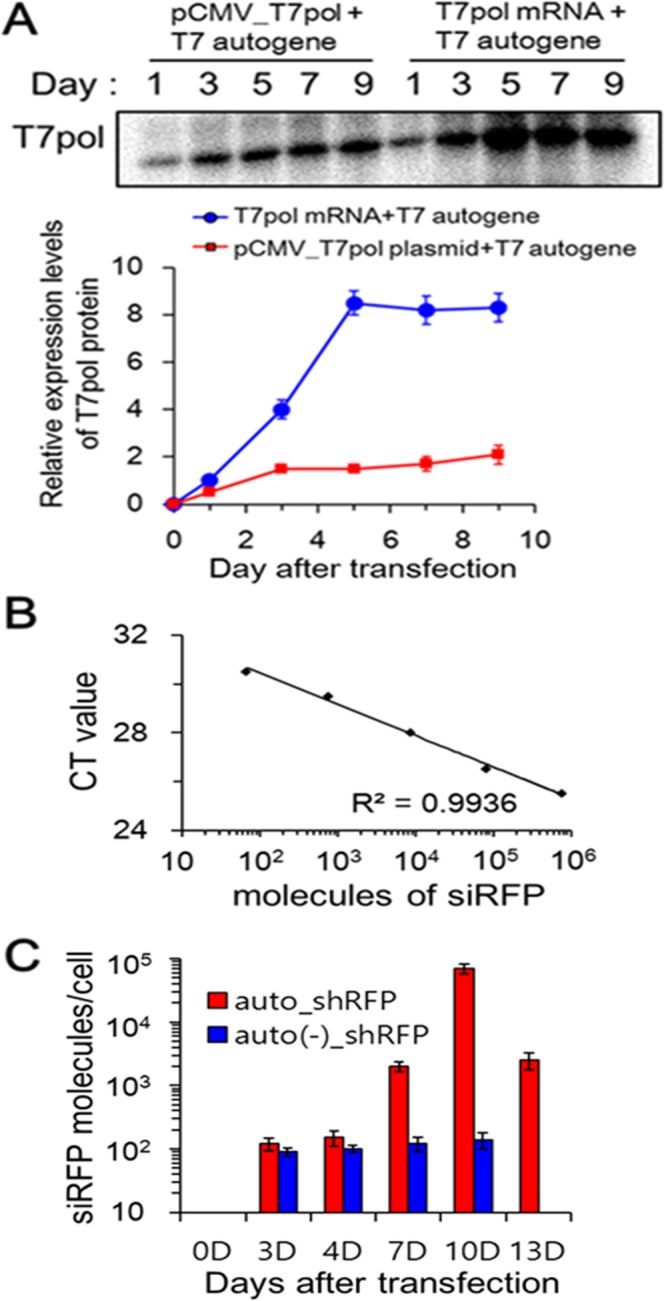
Sustained production of siRNA by higher and more sustained levels of T7 RNA polymerase. (**A**) Co-delivery of T7pol mRNA and T7 autogene induces higher and more sustained levels of T7pol expression than pCMV-triggering T7 autogene. B16F10/RFP cells were transfected by lipoplexes containing T7 autogene plasmids with either T7pol mRNAs or pCMV_T7pol plasmids for the specified period of time, and then cell lysates were subjected to immunoblot staining, based on the anti-T7 RNA polymerase polyclonal antibodies. Each band intensities were quantitatively analyzed using Image J software and EZ-Capture MG. Data represent the mean ± s.d. (n = 5). The full blot image is presented in Supplementary Fig. S4. (**B**) TaqMan standard curve of synthetic siRFP oligoes *versus* CT value. siRFP-specific small RNA TaqMan assay was carried out with serial dilution of synthetic siRFP oligoes. The number of siRFP molecules corresponding to each point was calculated and plotted with respect to CT value. (**C**) Quantitative measurement of siRFP molecules processed from shRFP hairpins. B16F10/RFP cells were transfected with auto_shRFP@LS or auto(−)_shRFP@LS for the indicated period of time, and then siRFP-specific small RNA TaqMan assay was performed against the RNA samples isolated from the cell lysates. Based on the standard curve in (**B**) and CT values obtained from siRFP amplification, the amounts of siRFP molecules produced could be calculated, and then plotted against incubation time after transfection. Data represent the mean ± s.d. (n = 5).

Previous dual promoters-driven T7-based (pCMV/pT7_T7pol) autogene has been reported to induce expression of T7pol for 5 days, where CMV promoter was used to trigger T7-based autogene plasmids after nuclear entry^27^. Here, we examined whether co-delivery of T7pol mRNA, instead of using CMV promoter for initial T7pol expression, could improve the efficiency of autocatalytic amplification of T7pol from T7 autogene. For this purpose, B16F10/RFP cells were transfected by lipoplexes containing T7 autogene plasmids with either T7pol mRNAs or pCMV_T7pol plasmids, where CMV promoter drives the transcription of T7pol in nucleus. Subsequently, each expression levels of T7pol in the cells were compared using a western blotting method and T7pol-specific antibodies. First, T7pol mRNAs/T7 autogene-transfected cells showed a significant increase in the level of T7pol proteins over time, which represents a hallmark of autocatalytic, self-amplification system (Fig. 3A). It was apparent that the co-delivery of T7pol mRNAs and T7 autogene maintained high levels of T7pol expression for least over 9 days. However, such an increase rate was much lowered in the pCMV_T7pol plasmids/T7 autogene-transfected cells, where the nuclear entry of plasmids was required for initial expression of T7pol. The ability of T7pol mRNA to improve the self-amplification efficiency of T7 autogene was evident in B16F10/RFP cells, and a 4.0-fold increase (*P* < 0.0001) in T7pol expression was observed for 9 days, when compared to that of pCMV-triggering T7 autogene. Because T7pol protein produced by translation of T7pol mRNA may induce efficient expression from co-delivered T7 autogene immediately without the need for mRNA amplification to occur, these results indicate that co-delivery of T7pol mRNA and T7 autogene can induce higher and more sustained expression of T7pol than the previous pCMV-triggering T7 autogene.

### Sustained production of siRNA *via* T7pol-driving shRNA expression

According to our shRNA expression strategy, self-replenished T7pol proteins enable pT7/shRNA DNA fragment to generate RNA hairpin as a premature form of siRNA. However, to suppress the expression of target gene, these hairpins should be processed into mature siRNA duplexes by the aid of cellular RNAi machinery. To quantitatively measure the production of mature RFP siRNA (siRFP) in the auto_shRFP@LS-transfected B16F10/RFP cells, we first created a standard curve of the guide strands of chemically synthesized siRFP oligoes by using a small RNA-specific TaqMan assay (Fig. 3B). In preparation of the TaqMan siRFP standard curve, the RFP-specific stem-loop reverse transcription (RT) primers provide specificity for guide strand in the RT reaction step, while the custom-made siRFP-specific primers with FAM-labeled fluorogenic probes offer specificity for RFP cDNA in the qPCR reaction step. This assay uses an exponential correlation between the amounts of siRFP oligoes and amplification pattern, but any amplification was not observed for either synthetic shRFP hairpin oligoes or DNA fragments such as pT7/shRFP DNA fragments (data not shown), which clearly indicates that the selected stem-loop RT primers are highly specific for the mature siRFP duplexes.

According to the standard curve, mature siRFP levels in the auto_shRFP@LS-transfected B16F10/RFP cells exponentially increased over time, reaching their maximum values 10 days post-transfection (Fig. 3C). In particular, it is noteworthy that a significant amount of mature siRFP remained after 13 days of transfection. However, auto(−)_shRFP@LS-transfected cells, which lacked T7 autogene plasmid, exhibited much lowered levels of mature siRFP, although there were linear increase over time. In the auto(−)_shRFP system, the source of T7pol is derived only from the initial supply of T7pol mRNA, and consequently the level of T7pol cannot be as high as that of auto_shRFP system. Therefore, these results indicate that although the conversion of shRNA into mature siRNA is required, the production of siRNA is proportional to the expression levels of T7pol.

### A more prolonged *in vitro* silencing effect of auto_shRFP@LS

**Figure 4:**
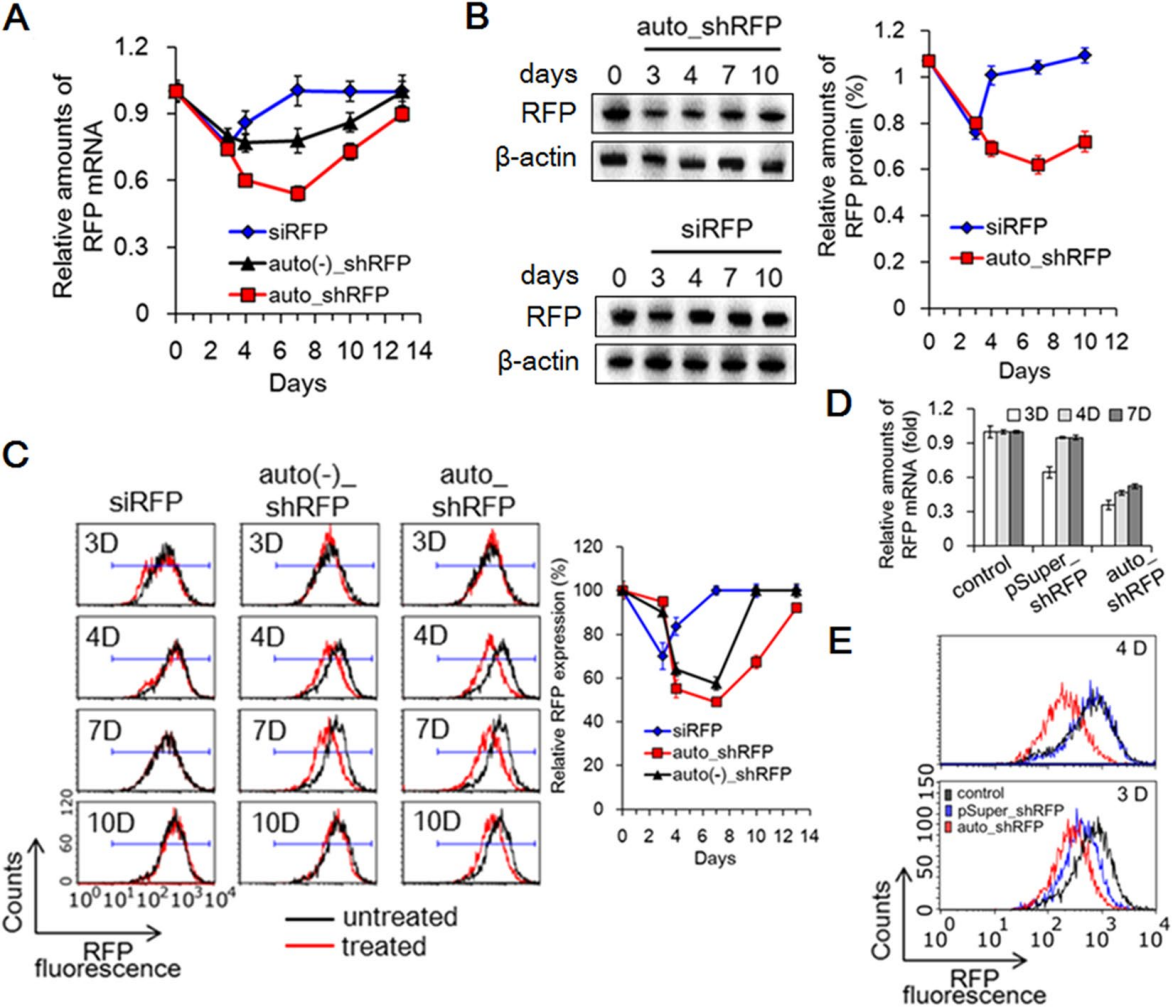
Prolonged silencing effects on target RFP gene in auto_shRFP@LS-transfected B16F10/RFP cells. (**A**) Sustained decrease of RFP mRNA in auto_shRFP@LS-transfected B16F10/RFP cells. For the indicated period of time, B16F10/RFP cells were transfected with synthetic siRFP@LS (corresponding to 50 nM of siRFP), auto_shRFP@LS (15 μg/mL), or auto(−)_shRFP@LS (14.04 μg/mL; corresponding to T7 autogene plasmid-lacking auto_shRFP system). For each RNA samples, the amounts of RFP mRNA were quantitatively estimated by qRT-PCR method, and then plotted against incubation time after transfection. For comparison, each amount of amplified RFP DNA products was normalized with respect to that of amplified β-actin DNA products. Data represent the mean ± s.d. (n = 5). (**B**) Sustained suppression of RFP expression in auto_shRFP@LS-transfected B16F10/RFP cells. After B16F10/RFP cells were transfected with auto_shRFP@LS or synthetic siRFP@LS for the specified period of time, the amounts of RFP protein within each protein samples were determined by immunoblotting method, and then plotted against incubation time after transfection. Data represent the mean ± s.d. (n = 5). The full blot images are presented in Supplementary Fig. S5. (**C**) Flow cytometry profiles demonstrating the sustained suppression of RFP expression. For the indicated period of time, B16F10/RFP cells were transfected in the same manner as (**A**). RFP-expressing cells were sorted with respect to a prefixed gate region for RFP fluorescence, and then their percentage was plotted against incubation time after transfection. Data represent the mean ± s.d. (n = 3). (**D**,**E**) A comparison of duration of silencing effect between auto_shRFP system and nuclear shRNA expression system. B16F10/RFP cells were transfected with auto_shRFP@LS (15 μg/mL) or pSuper_shRFP plasmid@LS (15 μg/mL; corresponding to nuclear H1 promoter-driven shRFP expression system), and then relative amounts of RFP mRNA within the cells were plotted *versus* transfection time (**D**). The representative FACS profiles on 3 days and 4 days post-transfection are presented (**E**).

To investigate whether the duration of silencing effects in eukaryotic cells could be prolonged, we quantitatively assessed the silencing effects of hybrid system on RFP target gene over time. A main characteristic of RNAi function is a substantial decrease in the post-transcriptional level due to degradation of target mRNA. Thus, the levels of RFP mRNA were quantitatively measured in the auto_shRFP@LS-transfected B16F10/RFP cells by using qRT-PCR method. As a control, the exogenous siRFP@LS-transfected B16F10/RFP cells showed that RFP mRNA levels remained decreased for at least 4 days after transfection, while reaching their minimum value of 75% 3 days post-transfection (Fig. 4A). Their RFP mRNA levels completely restored 7 days post-transfection. However, the auto_shRNA@LS-transfected B16F10/RFP cells revealed that RFP mRNA levels steadily decreased over a longer period of time (for at least 13 days), and particularly, reached a minimum of 54% 7 days post-transfection. Auto(−)_shRFP system also lead to a continuous decrease in RFP mRNA levels over 10 days after transfection, but its efficiency for destruction of mRNA was much lower than that seen with auto_shRFP system, as expected.

To further examine the longevity of silencing effects *in vitro*, the levels of RFP protein were monitored by using a western blotting assay and RFP-specific antibodies. The auto_shRFP@LS-transfected B16F10/RFP cells showed that there was a significant reduction in RFP protein levels for at least 10 days after transfection (Fig. 4B). However, the reduction of RFP protein seen with exogenous siRNA method was much smaller than that seen with auto_shRNA system, and lasted only for 3 days after transfection, as previously shown in the most conventional siRNA methods.

Consistently with these results, flow cytometry measurement also demonstrated that the RFP fluorescence signals of auto_shRFP@LS-transfected B16F10/RFP cells maximally decreased down to 49% 7 days post-transfection, and even 10 days after transfection, approximately 33% of decrease was still seen (Fig. 4C). However, the exogenous siRFP and auto(−)_shRNA system showed much weaker inhibitory effects on RFP fluorescence signals over a relatively short period of time, when compared to the auto_shRFP system. Taken together, these results indicate that T7 autogene-based hybrid system, upon employed together with the nonviral liposomal vehicles, can significantly enhance the duration of silencing effects on the target gene in eukaryotic cells.

### Cytoplasmic auto_shRNA system *vs* nuclear shRNA expression system for durable gene silencing

To compare the relative silencing durations of T7-based autogene hybrid system and nuclear shRNA expression system, we prepared H1 promoter-driven shRFP expression plasmid (pSuper_shRFP), which can produce nuclear shRFP transcripts with two 3′ overhanging U nucleotides using nuclear transcription machinery (Supplementary Fig. S1). On each B16F10/RFP cells transfected with the same amounts of pSuper_shRFP@LS and auto_shRFP@LS, the relative amounts of RFP mRNA were measured at the indicated time points by using qRT-PCR method. When monitored over time, the reduced RFP mRNA levels in the pSuper_shRFP-transfected cells lasted for only 3 days, whereas much more reduced mRNA levels in the auto_shRFP-transfected cells persisted for more than 7 days (Fig. 4D). In particular, the semi-quantitative comparison of RFP mRNA levels 3 days post-transfection revealed that auto_shRFP system could result in a 1.8-fold reduction of mRNA over pSuper_shRFP system. Flow cytometry analysis also showed that the duration of RFP gene suppression in the auto_shRFP-transfected cells was longer than in the pSuper_shRFP-transfected cells, as expected (Fig. 4E). RFP fluorescence signals of auto_shRFP@LS-transfected cells were still suppressed 4 days post-transfection, whereas those of pSuper_shRFP@LS-transfected cells were completely restored. Thus, these results indicate that our T7 autogene-based hybrid system can provide a more efficient means for both prolonged duration and efficiency of gene silencing than nuclear shRNA expression system.

### *In vivo* biodistribution studies of auto_shRFP@LS in tumor xenograft mice

**Figure 5:**
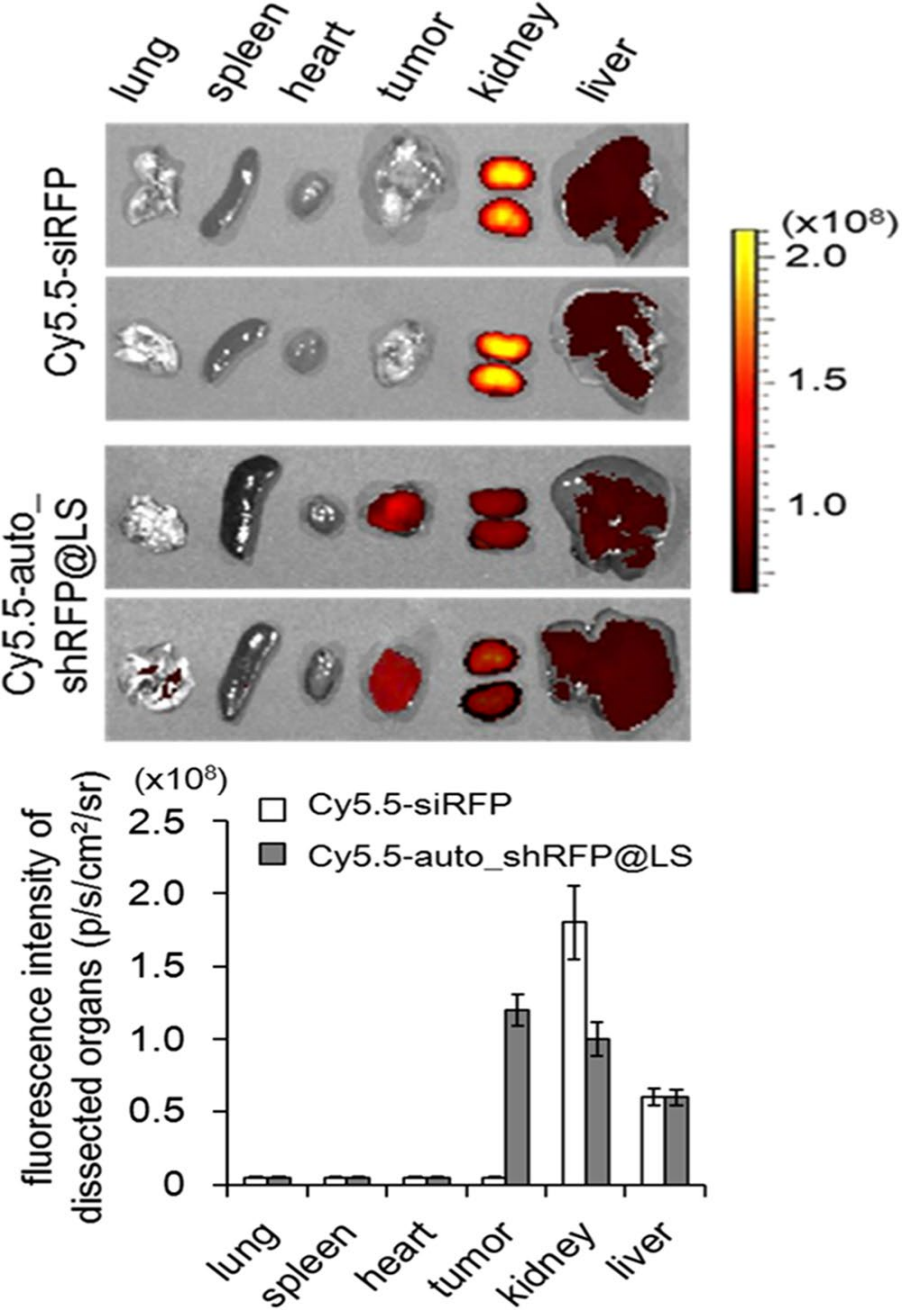
Biodistribution of auto_shRFP@LS in B16F10 tumor-bearing mice. *Ex vivo* fluorescence images of the tumors and organs dissected from euthanized mice 24 h after a single intravenous administration of Cy5.5-labeled auto_shRFP@LS or Cy5.5-labeled free siRFP were measured by IVIS Spectrum. Fluorescence intensities of Cy5.5-labeled auto_shRFP@LS or Cy5.5-siRFP that accumulated at the major vital organs and tumors were plotted below. Data represent the mean ± s.d. (n = 3 mice per group).

To examine whether auto_shRFP@LS could extravasate from blood vessels to the sites of tumor while relying on the enhanced permeability and retention (EPR) effect, Cy5.5-labeled auto_shRFP@LS or free Cy5.5-siRFP were intravenously administered into B16F10 xenograft mice. When major vital organs and tumors were dissected 24 h post-injection to measure near-infrared fluorescence images, their *ex vivo* images from free Cy5.5-siRFP-injected mice, as a control, showed that there existed no fluorescence intensity at the tumors, while strong fluorescence intensities were observed predominantly in the kidneys (Fig. 5). However, the *ex vivo* images from Cy5.5-auto_shRFP@LS-injected mice exhibited the strong fluorescence intensities at tumors, although non-negligible amounts of Cy5.5-auto_shRFP@LS were still seen in the kidneys and livers. These results indicate that DOPC liposomes loaded with hybrid mRNA/DNA can preferentially accumulate in the tumor sites through the EPR effect highly associated with the particle size (about 31 nm).

### *In vivo* gene silencing effects of auto_shRFP@LS on tumor xenograft mice

**Figure 6:**
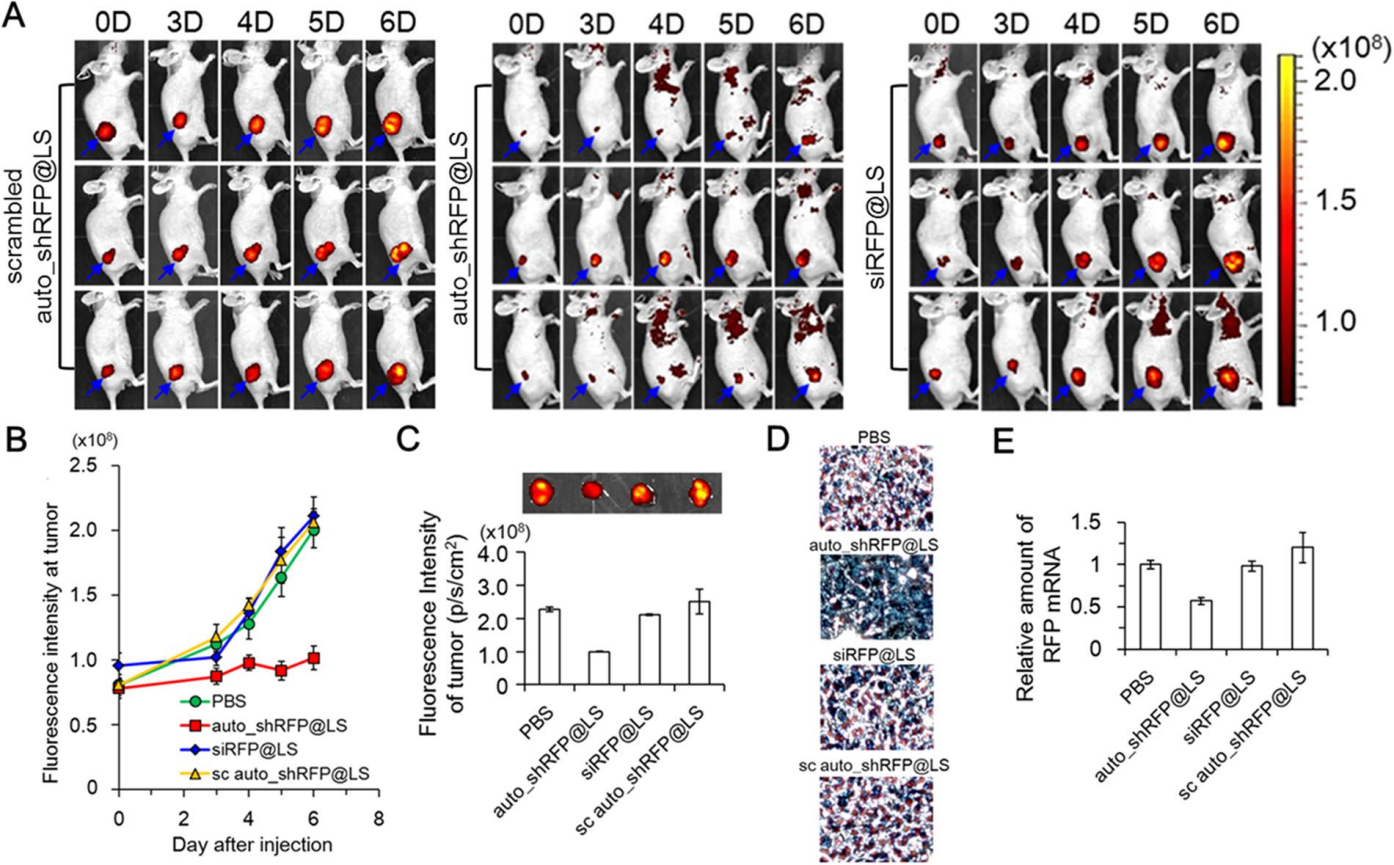
Prolonged *in vivo* RFP gene silencing effects of auto_shRFP@LS on B16F10/RFP tumor-bearing mice. (**A,B**) Noninvasive fluorescence images of RFP-expressing tumor sites from auto_shRFP@LS-injected mice and their fluorescence intensities. Tumor sites are indicated by blue arrows. B16F10/RFP tumor-bearing mice were intravenously injected with auto_shRFP@LS, PBS, scrambled auto_shRFP@LS or synthetic siRFP@LS only once (day 0; 40 μg per injection). At the specified time points (0, 3, 4, 5, and 6 days), RFP fluorescence intensities on the whole bodies were measured by IVIS Spectrum (**A**), and then those at the tumor sites were plotted against day after injection (**B**). Data represent the mean ± s.d. (n = 3 mice per group). The noninvasive fluorescence images of PBS-injected mice are presented in the Supplementary Fig. S2. (**C**) *Ex vivo* RFP fluorescence images of the dissected tumors 6 days post-injection. RFP fluorescence intensities of the dissected tumors were analyzed by IVIS Spectrum, and then plotted (n = 3 mice per group). (**D**) Immunohistochemical analysis of the dissected tumors using anti-RFP antibodies. Anti-RFP antibodies are indicated by brown spots. (**E**) Quantitative RT-PCR results of RFP mRNA in the excised tumors. The relative levels of RFP mRNA were compared by normalizing with those of β-actin mRNA. Data represent the mean ± s.d. (n = 5).

Using a fluorescence animal imaging system, we examined the efficiency and duration of *in vivo* gene silencing in the auto_shRFP@LS-injected B16F10/RFP-bearing mice. According to the previous *in vivo* RNAi studies, siRNA formulations should be injected every 2 or 3 days to maintain the silencing effects due to their short duration of silencing effects^3^. In the current studies, each mouse, however, intravenously received auto_shRFP@LS or siRFP@LS only once before measuring the relative *in vivo* durations of silencing effects. As a control, scrambled auto_shRFP@LS-injected mice clearly showed that as the B16F10/RFP tumors grew, the fluorescence intensities of RFP signals at tumor sites increased proportionally (Fig. 6A). However, auto_shRFP@LS-injected mice showed that RFP signals at tumor sites were significantly suppressed during the six-day monitoring period, although the tumor sizes were steadily increasing. Synthetic siRFP@LS-injected mice showed a slowing of increase in the RFP signals for the first three days, but since then, ultimately revealed a sharp increase, leading to the similar levels of RFP fluorescence intensities to those from scrambled auto_shRFP@LS-injected mice (Fig. 6B). The PBS-injected mice showed the proportional increase of RFP fluorescence intensity to the tumor growth, as expected (Supplementary Fig. S2). Compared to each *ex vivo* fluorescence images of tumor tissues excised from the PBS-injected, synthetic siRFP@LS-injected, or scrambled auto_shRFP@LS-injected mice, auto_shRFP@LS-injected mice revealed a significant reduction of RFP signals, where the RFP total photon counts per gram of tumor were 2.7-fold lower than those from the PBS-injected mice (Fig. 6C).

Immunohistochemical staining assay, based on RFP-specific antibodies, also demonstrated a large quantity of RFP proteins located in the excised tumors from the PBS-injected, siRFP@LS-injected, or scrambled auto_shRFP@LS-injected mice, but not from the auto_shRFP@LS-injected mice (Fig. 6D). When the amounts of RFP mRNA, isolated from the excised tumor tissues 6 days post-injection, were quantitatively estimated by qRT-PCR analysis, the auto_shRFP@LS-injected mice showed about 43% of decrease in RFP mRNA levels compared to those of the PBS-injected mice (Fig. 6E). On the other hand, either siRFP@LS-injected or scrambled auto_shRFP@LS-injected mice did not show any reduction in RFP mRNA levels. Taken together, these results indicate that our T7 autogene-based hybrid system induced the long-term silencing of target gene on tumor sites *in vivo* without the repeated injections.

Finally, we examined the innate immune response of auto_shRFP@LS by monitoring the released TNF-α and INF-α on blood samples (Supplementary Fig. S3). When systemically administered, either auto_shRFP@LS or scrambled auto_shRFP@LS did not cause any appreciable elevation of TNF-α in serum both 1 h and 24 h post-injection, respectively. However, siRFP@lipofectamine, corresponding to the conventional *in vitro* transfection reagent/siRNA complexes^28^, induced the remarkable elevations of TNF-α in serum 1 h post-injection, although we did not observe such an elevation anymore 24 h post-injection. Also, siRFP@lipofectamine showed the similar mode in the release of INF-α, in that the significant increase in serum 1 h post-injection returned to the basal levels 24 h post-injection. But, auto_shRFP@LS did not cause any appreciable INF-α release at both time points.

## Discussion

The main disadvantage of synthetic siRNA in the therapeutic use is the transient silencing ability, owing to the biodegradation, dilution upon cell division, and its nonrenewable property^29^. To achieve a consistent gene silencing effect *in vivo*, repeated administration of siRNA is thus required on a prolonged period of time. However, frequent administration strategies for siRNA therapeutics have been a major impediment to progress in the development of RNAi therapies, as shown in the clinical trials so far^30^.

Here, we demonstrated that the nonviral, hybrid mRNA/DNA system, composed of T7pol mRNA, pT7/shRNA DNA fragment, and T7 autogene plasmid, could express shRNA in cytoplasm for a prolonged period of time, ultimately generating siRNA for more than 13 days through the function of cellular RNAi machinery. Finally, liposome-mediated systemic administration of this hybrid system into tumor-bearing mice showed that target RFP gene at the tumor sites could be successfully silenced with a single intravenous injection, in contrast to the conventional siRNA methods relying on the repeated injections every 2 or 3 days. To our knowledge, this study shows the first evidence that T7 autogene-based cytoplasmic system for shRNA expression can induce the long-term silencing *in vivo*.

The low levels of transfection efficiency that can be achieved by current nonviral gene delivery technologies have limited the use of plasmid-based shRNA expression system. In addition, highly expressed shRNA in nucleus are likely to induce saturation of exportin-5 transporter through competition with microRNA for nuclear export, potentially result in the unexpected toxicities^31^. One approach to address these problems is to design a cytoplasmic shRNA expression system that does not require either nuclear entry of genes for shRNA expression to occur or nuclear export of produced shRNA, and thus our cytoplasmic shRNA expression system suggests an efficient strategy to address the obstacles potentially caused by plasmid-based shRNA expression system.

Well-established viral vector systems such as adenovirus, lentivirus, and adeno-associated virus are already available for long-term expression of shRNA. However, these recombinant viral vectors have the risk of potential mutagenesis and immunogenicity in the infected body as well as the high cost for high-titer viruses, and thus, their clinical applications have been limited. Herein, our T7 autogene-based hybrid mRNA/DNA system provides an efficient means to enable the long-term expression of shRNA without such limitations.

Originally, T7-based autogene systems have been used to produce high levels of proteins from pT7-driven transgene because the repeated expression of T7pol through an autocatalytic positive feedback loop can generate a large amount of T7pol proteins^19^. However, in contrast to the increased protein yields, there is still a difficulty in extending the duration of transgene expression by T7-based autogene. One of the prerequisites for achieving the sustainable shRNA expression under the control of T7 promoter was a design of T7 autogene system that could stably supply T7pol proteins for a prolonged period of time. Here, our significant finding was that co-delivery of T7pol mRNA and T7 autogene could improve the autocatalytic efficiency of T7 autogene by a 4.0-fold for at least 9 days, compared to the previous use of CMV promoter for initial T7pol expression in the pCMV-triggering T7 autogene^27^. That is, the co-delivered T7pol mRNAs was able to trigger T7 autogene immediately without mRNA amplification process, as well as to bypass the nuclear barrier problems.

In the current studies, we have observed that co-delivery of T7pol mRNA and IRES-lacking T7 autogene, where cytoplasmic T7pol transcripts lack IRES elements, lead to only a small amount of T7pol proteins in B16F10/RFP cells (data not shown). These results indicate that although T7pol expression was triggered by exogenous T7pol mRNA, most cytoplasmic T7pol transcripts from IRES-lacking T7 autogene were rarely translated into T7pol protein due to the lack of IRES element. Thus, the use of IRES elements is likely to improve the autocatalytic efficiency of T7 autogene, together with the co-delivered T7pol mRNA. Although viral IRES elements have been incorporated to the previous T7-based autogene systems for enhancing the translational efficiency of T7pol gene, a eukaryotic promoter upstream of IRES elements required the nuclear entry of autogene plasmids for induction of early T7pol expression^22^.

To improve the duration of siRNA-mediated silencing, there have been extensive works to increase the cargo capacity for siRNA by developing a variety of delivery vehicles such as polymers^32^, dendrimers^33^, and liposomes^34^. But, these attempts have faced the unwanted problems including the use of large amounts of synthetic polycationic reagents such as polyethylenimine, which potentially cause undesirable cytotoxicity, immunogenicity or nonspecific accumulation in body^35^. Also, low density of negative charge and intrinsic stiffness on siRNA oligoes are unfavorable to promote siRNA loading^36^. Because our T7 autogene-based hybrid mRNA/DNA system exploits a strategy to sustainably express high levels of shRNA rather than to acquire high cargo capacity for siRNA, it can avoid the use of an overdose of polycationic reagents and thus would be able to reduce the potential risk of cytotoxicity and immunogenicity.

The transient transfection of siRNA has usually variation in transfection efficiency, and as a result, siRNA-mediated inhibition of specific genes has been limited in the applications of difficult-to-transfect cell lines including primary immune cells^37^. Also, a single transfection of siRNA is not sufficient to offer a sufficient time window of functional inhibition when target proteins have long half-lives. Therefore, the sustainable expression capability of T7 autogene-based hybrid system is expected to overcome these limitations in the applications of RNAi.

Considering the clinical application of T7 autogene-based hybrid mRNA/DNA system, adaptive immunity may abolish transgene expression of T7pol proteins in the body. This immune response may make RNAi therapy ineffective and cause severe inflammation if immune system eliminates the transfected cells. Thus, it would be necessary to determine the extent to which transgene expression of T7pol proteins results in serious immunogenicity problems. As an alternative to the clinical setting, the combination with immunosuppressive agents as adjuvants may be effective in avoiding such immune responses, as shown in the treatment of transplantation patients with immunosuppressive agents^38^.

It has proven difficult to systemically deliver nucleic acids to tumors other than liver due to the nonspecific uptake by reticuloendothelial system and insufficient transfection in the tumor cells^39^. We demonstrated that T7 autogene-based hybrid mRNA/DNA system could be delivered to tumor sites *in vivo* by the aid of DOPC liposomal vehicle. This finding thus speculates that extravasation of lipoplexes into the interstitial space occurred by the enhanced permeability and retention (EPR) effect, and eventually lead to tumor-specific accumulation. EPR effect involves the altered biodistribution, where the nano-sized lipoplexes show the significant accumulation in the tumor sites reaching higher concentration than other organs^40^. Moreover, the particle size of lipoplexes is expected to exceed the limit of renal excretion threshold, which reduces the renal clearance and thus increases their plasma half-life.

Recently, we have examined the therapeutic availability of our T7 autogene-based hybrid system using the SKOV3 tumor-bearing mice. When EGFR shRNA sequences were applied to pT7/shRNA DNA fragments, a single systemic injection could suppress the expression of EGFR gene in the tumor sites for more than two weeks, substantially inhibiting the ovarian tumor growth. These preliminary studies suggest that our T7 autogene-based hybrid system is not limited to either specific target gene or specific cell line, and can be extended to cancer therapies that require the long-term suppression of tumor-associated genes. Particularly, our hybrid system as siRNA therapeutics suggests a lot of benefits in the treatment of chronic diseases including scaling down the administration frequency in dosing schedule.

In summary, we demonstrated that the development of T7 autogene-based hybrid mRNA/DNA system for sustainable shRNA expression in cytoplasm could address the limitations caused by plasmid-based shRNA expression system. Furthermore, these studies imply that our hybrid mRNA/DNA system has great potentials to serve as a nonviral platform technology for long-term silencing effects *in vitro* and *in vivo*.

## Methods

### Materials

1,2-dioleoyl-sn-glycero-3-phosphatidylcholine (DOPC) and all other organic reagents were obtained from Sigma Aldrich (St. Louis, US). Cell culture media were purchased from WelGENE (Daejeon, Korea). RFP siRNAs were synthesized by Bioneer (Korea) as following sequences: 5′-UGUAGAUGGACUUGAACUCdTdT-3′ (sense) and 5′-GACUUCAAGUGCAACUUCAdTdT-3′ (anti-sense). Cy-5.5-labeled RFP siRNA (Cy-5.5 dye is conjugated to 5′-end of sense strand) was also purchased from Bioneer. For determination of RFP and mouse β-actin mRNA levels, primers were also synthesized by Bioneer as following sequences: for RFP mRNA, 5′-GGCTGCTTCATCTACAAGGT-3′ (forward) and 5′-GCGTCCACGTAGTAGTAGCC-3′ (reverse); for mouse β-actin mRNA, 5′-GGCTGCTTCATCTACAAGGT-3′ (forward) and 5′-ACATCTGCTGGAAGGTGGAC-3′ (reverse). For the control silencing experiments, all the siRNA oligoes were administered into cells at a final concentration of 50 nM. The primary antibodies against RFP and β-actin were obtained from Santa Cruz Biotechnology (Santa Cruz, US) and Sigma Aldrich, respectively. The secondary antibodies were obtained from Bethyl Laboratories (Montgomery, US). The mouse melanoma B16F10 and B16F10/RFP cell lines were purchased from Korean Cell Line Bank (Seoul, Korea).

### Construction of T7 autogene plasmid

T7pol gene (GenBank accession no. FJ881694.1) with the overlapping BamHI/XhoI site sequences was amplified against chromosomal DNA of *E. coli* BL21 (DE3) (Novagen, US) as a DNA template by using PCR reaction: the primer sequences used for amplification are 5′-GGTCGCGGATCCATGAACACGATTAACATCGCT-3′ (forward) and 5′-GTGCTCGAGTTACGCGAACGCGAAGTCCGACTC-3′ (reverse), where the target sequences of T7pol is underlined. The resulting PCR fragments were digested using BamHI/XhoI enzymes, and then ligated into the multi-cloning sites of pT7CFE1-NHis plasmid (Thermo Fisher, US), which are located downstream to the cognate T7 promotor and EMCV IRES sequences and upstream to the polyA sequence and T7 terminator. The resulting T7 autogene plasmids were verified by DNA sequencing. To achieve CMV promoter-driven expression of T7pol, we also cloned T7pol full-length genes as BamHI-XhoI fragments into pFLAG-CMV-2 plasmids (Sigma Aldrich) in the same manner as described above. The resulting pCMV_T7pol plasmids contained T7pol gene under control of CMV promoter.

Additionally, IRES-lacking T7 autogene plasmids were constructed from T7 autogene plasmids using QuickChange site-directed mutagenesis kit (Agilent Technologies, US). Briefly, 45-bp of synthetic primers that connected the flanking regions of IRES sequence to be deleted were prepared, and then extended against T7 autogene plasmids through *PfuTurbo* DNA polymerase-mediated temperature cycling. The resulting mutated plasmids with the staggered nicks were treated with *Dpn* I endonuclease to remove the parental DNA templates, and then XL1-Blue competent cells were transformed with the nicked DNA plasmids containing the deletion mutation. The IRES-lacking T7 autogene plasmids prepared from the blue colonies were verified by DNA sequencing.

### Synthesis of pT7/shRFP DNA fragment and 5′-capped T7pol mRNA

pT7/shRFP DNA fragments were generated by assembling the short fragments through DNA hybridization and annealing, according to an assembly PCR method^41^. Briefly, the oligoes were designed to be either part of top or bottom strand of pT7/shRFP DNA fragment. During the PCR reaction, the oligoes were annealed to the complementary fragments, and subsequently were filled-in by the function of DNA polymerase. After PCR products were purified using PCR purification Mini kit (Favorgen, Taiwan), the complete sequences were confirmed on agar gel electrophoresis. The sequences of the resulting pT7/shRNA DNA fragments, when verified by DNA sequencing, were as following: 5′-taatacgactcactatagg GAGTTCAAGTCCATCTACATTCAAGAGATGTAGATGGACTTGAACTC-3′, 5′-**GAGTTCAAGTCCATCTACA**TCTCTTGAA**TGTAGATGGACTTGAACTC**tatagtgagtcgtatta-3′, where the T7 promoter sequences are indicated in lowercase, the RFP siRNA sequences are in bold, and the loop sequences are underlined. Noticeably, the first nucleotide was designed to be guanosine, because the efficient T7pol initiation requires the first nucleotide of RNA to be guanosine. Synthesis of scrambled siRFP-containing pT7/shRFP DNA fragments was carried out in a similar way to that of pT7/shRFP DNA fragments by using the mismatched siRFP sequences. Also, Cy5.5-dUTP-incorporated pT7/shRFP DNA fragments, required for the preparation of Cy5.5-labeled lipoplexes, could be synthesized by using PCR reaction with proper primer pairs and a 4:1 mixture of dTTPs and Cy5.5-dUTPs (Enzo, US).

To synthesis 5′-capped T7pol mRNA, chemically synthesized dsDNA templates, designed to locate T7 promoter sequences upstream of T7pol-encoding gene, were first obtained from Bioneer, and then *in vitro* synthesis of 5′-capped T7pol mRNA was carried out using HiScribe T7 High Yield RNA Synthesis Kit (NEB, US). Anti-reverse cap analog (ARCA), also known as 3′-O-Me-7mG(5′)ppp(5′)G cap analog, was incorporated into the *in vitro* transcription reaction, along with the four standard NTPs, in a ratio of cap analog to GTP 4:1. DNA nucleases were added to remove the dsDNA templates, and then the resulting 5′-capped mRNA was purified by LiCl extraction and ethanol precipitation methods. As a control, a standard, 5′-cap-free T7pol mRNA synthesis was carried out by using the same dsDNA templates and MEGAscript T7 Transcription Kit (Thermo Fisher Scientific, US). NanoDrop™ (Thermo Fisher Scientific, US) was used to measure the concentration of mRNA.

### Construction of nuclear shRNA expression plasmid

To construct pSuper_shRFP plasmids for nuclear transcription of RFP shRNA, pSuper basic plasmids (OligoEngine, US) were digested with BglII and XhoI restriction enzymes, and then the annealed oligoes were inserted into the plasmids using T4 DNA ligase: 5′-GGATCCCCGAGTTCAAGTCCATCTACAttcaagagaTGTAGATGGACTTGAACTCTTTTTCTCGAG-3′ and 5′-CTCGAGAAAAAGAGTTCAAGTCCATCTACAtctcttgaaTGTAGATGGACTTGAAGTCGGGGATCC-3′, where the RFP siRNA sequences are underlined and loop sequences are in lowercase. It is a noted fact that when these plasmids produce short hairpin RNA transcript, they use RNA polymerase III H1 promoter. The resulting shRNA has a well-defined start of transcription and a termination signal consisting of five thymidines (T5). At the termination site, the cleavage of transcripts occurs after the second uridine^42^, resulting in a short hairpin RNA with 19-bp stem region, 9-nt loop region and two 3′ overhanging U nucleotides. The resulting pSuper_shRFP plasmids were finally verified by DNA sequencing.

### Preparation of lipoplexes

Liposome-formulated lipoplexes (auto_shRNA@LS) were prepared by mixing the neutral lipid DOPC (26.5 μg) with pT7/shRNA DNA fragment (0.15 μg, 2.5 pmol), 5′-capped T7pol mRNA (0.625 μg, 0.625 pmol), and T7 autogene plasmid (1.875 μg, 0.625 pmol) in the presence of excess *t*-butanol, as previously described^25^. After Tween 20 was added (Tween 20:oligonucleotides/DOPC = 1:19, w/w), the mixture was lyophilized in an acetone/dry ice bath to remove the residual organic solvent. Next, normal 0.9% saline solution was added to hydrate this lyophilized mixture, and then free oligonucleotides, not taken up by liposomes, were removed by using Amicon ultracentrifugal filter (30 K MWCO, Millipore). The concentration of free oligonucleotides passed through a filter was measured by Nanodrop spectrophotometer, and finally the complexing efficiency of oligonucleotides with liposomes was determined as more than 90%. The fractions trapped in the filter were reconstituted with 0.9% saline until reaching at the final concentration of 150 μg/mL. For a preparation of fluorescent lipoplexes, Cy5.5-labeled pT7/shRNA DNA fragments were additionally mixed to hybrid mRNA/DNA components at a weight ratio of 0.5%.

### Physichochemical characterization of lipoplexes

The hydrodynamic diameter and zeta potential of lipoplexes was measured by Zetasizer Nano ZS (Malvern, UK) and Zetasizer Nano software. All samples were filtered with a syringe filter (MWCO = 0.22 μm), serially diluted with nuclease-free water, and then their data were collected at a scattering angle of 173° with a standard 633 nm laser. While a fixed amount of oligonucleotides were complexed with liposomes at the specified weight ratio, gel retardation assay was carried out to evaluate the complexing ratio between oligonucleotides and liposomes. The oligonucleotides/DOPC samples were loaded on 1.5% agarose gel, and then their bands could be visualized under UV irradiation.

### Cell culture and transfection

Mouse B16F10 melanoma cells were seeded in 96-well plates (2 × 10^4^ cells/well) in DMEM media supplied with 10% FBS, 25 mM HEPES buffer, 100 U/ml penicillin and 100 mg/mL streptomycin in a humidified atmosphere of 5% CO_2_ at 37 °C, and then cultured overnight to reach 70% confluency. Similarly, RFP-expressing B16F10 (B16F10/RFP) mouse melanoma cells were cultured in RPMI 1640 media supplied with 10% FBS and 500 ng/mL G418. Immediately before transfection, the media were replaced with FBS-free media, and then lipoplexes solutions were transfected to each well at the final concentration of 15 μg/mL for 2 h. After the media was washed three times with fresh media including 10% FBS, the cells were further cultured for the specified period of time.

### Cell viability test

Cell viability of lipoplexes was determined using an MTT assay kit (Biosesang, Korea) as described previously^43^. Briefly, B16F10/RFP cells were seeded on each well of a 96-well plate at a cell density of 3 × 10^3^ cells/mL and allowed to further grow for 24 h. Various doses of lipoplexes were mixed into each well in a dose-dependent way. At 2 h post-transfection, the cells were washed three times using fresh media including 10% FBS, and subsequently further incubated for 22 h or 46 h. Next, MTT solution was supplemented to each well, and then further incubated for 2 h at 37 °C to generate formazan crystals. Immediately after the resulting crystals were dissolved in Sorensen’s glycine buffer and DMSO, the quantity was determined at 570 nm using VersaMax microtiter plate reader (Molecular Devices, US).

### Examination of TNF-α and INF-α release

Following a standard Ficoll-Paque density-gradient centrifugation method, we isolated peripheral blood mononuclear cells (PBMCs) from human peripheral blood as previously described^44^. First, we mixed anticoagulant-treated blood with an equal volume of PBS. For isolation of mononuclear cells, we added 10 mL of Lymphoprep solution to a 50-mL centrifuge tube, and then carefully layered the prepared blood sample onto the Lymphoprep solution. After centrifuging the tube at 400 g for 30 to 40 min at 16 °C, we drew off the upper layer corresponding to plasma and platelets using a pipette. The layer of monoculcear cells were transferred to a new tube using a pipette. After washing with 3 volumes of PBS buffer, the mononuclear cells were collected by centrifuging. Next, we seeded the isolated PBMCs into 96-well plates at density of 2 × 10^4^ cells/well in RPMI 1640 containing 10% FBS and 2 mM L-glutamine. For TNF-α and INF-α analysis, we stimulated the PBMCs with the following stimuli: PBS (5 μM), auto_shRFP@LS (15 μg/mL), and lipopolysaccharides (55 ng/mL) for TNF-α or CpG oligodeoxynucleotides (6 μM) for INF-α. Lipopolysaccharide and CpG oligodeoxynucleotide was used as a representative stimulus for each of TNF-α and INF-α. At the specified time points such as 4 h and 24 h post-transfection, we quantitatively measured the released TNF-α and INF-α from the collected supernatants by using sandwich ELISA kit (Abcam, US) and VeriKine Human INF-alpha ELISA kit (PBL Biomedical, US), respectively.

For the *in vivo* immunogenic studies, we analyzed the plasma TNF-α and INF-α levels in female C57BL/6J mice. We intravenously injected mice with the following stimuli: PBS (5 μg per injection), siRFP@Lipofectamine (54 μg per injection) or auto_shRFP@LS (54 μg per injection). PBS buffer as a mock was injected into the control mice. At the specified time points such as 1 h and 24 h post-injection, plasma TNF-α and INF-α levels were analyzed from the blood samples with TNF-α-Ab ELISA kit (Thermo scientific, US) and INF-α-Ab ELISA kit (eBioscience, US), respectively.

### Western blots for quantification of endogenously produced T7pol proteins

B16F10/RFP cells were transfected by lipoplexes containing T7 autogene plasmids with either T7pol mRNAs or pCMV_T7pol plasmids. Next, total proteins were extracted from the transfected cell lysates at the indicated time points (1, 3, 5, 7, and 9 days) by using RIPA buffer (Sigma, US) containing cocktail protease inhibitors (Roche, Germany). After centrifugation, the collected protein samples were subjected to electrophoresis on a 10% SDS-PAGE gel, and then transferred to PVDF (polyvinyl difluoride) membranes. These membranes were blocked with PBS buffer including 0.2% Tween-20 and 5% dry milk, stained with anti-T7 RNA polymerase polyclonal antibodies, and finally incubated with HRP-conjugated secondary antibodies according to an enhanced chemiluminescence system (Abclon, Korea). The resulting band intensities were quantitatively measured by using Image J software and EZ-Capture MG (Japan).

### Quantification of mature siRNA using TaqMan assay

First, reverse transcription reactions for RFP siRNA was carried out using the Custom TaqMan® Small RNA assay kit (Applied Biosystems, US). Briefly, 2 μg of RNA was isolated and purified from B16F10/RFP cells transfected with either auto_shRFP@LS (15 μg/mL) or auto(−)_shRFP@LS (14.04 μg/mL), and then its cDNA was synthesized using custom-made siRFP-specific stem-loop TR primers. Next, quantitative PCR (qPCR) reactions were performed using cDNA and custom-made siRFP-specific primers with the fluoroscent FAM-abeled probes, which were obtained from Applied Biosystems (Assay ID: CT32Z7Y). β-actin gene was amplified for a loading control. The guide strands of the synthetic siRFP oligoes were used to generate a TaqMan standard curve: the sequence of guide strand is 5′-GAGUUCAAGUCCAUCUACA-3′. Finally, based on the amplification plot of a dilution series of synthetic siRFP standards, siRFP molecule number per cell could be estimated.

### Intracellular uptake studies of lipoplexes

Auto_shRFP@LS lipoplexes containing additional Cy5.5-labeled pT7/shRNA DNA fragments were transfected to B16F10 cells in 12-well plates at the final concentration of 15 μg/mL for 2 h, and the cells were washed three times with fresh PBS buffer. The cells were further incubated for 22 h and then detached from well plates by using trypsine-EDTA reagents. After the collected cells (2 × 10^5^ cells) were washed with DPBS including CaCl_2_ and MgCl_2_, the cells were sorted by Guava easyCyte flow cytometry system (Millipore, US) equipped with a red laser (excitation at 635 nm and emission at 665 nm). Additionally, confocal microscopic images were investigated by a Carl Zeiss LSM 700 microscope (Carl Zeiss, Germany) equipped with 514 nm laser for Cy5.5 fluorescence, while the images were processed and quantified by ZEN 2012 software. The nuclei were counterstained with DAPI.

### qRT-PCR and immunoblotting for quantitative determination of RFP mRNA and protein

For qRT-PCR reactions, total RNA samples were first extracted from the cell lysates by using Trizol reagent (Invitrogen, US) and the complementary DNA (cDNA) was reversely transcribed by using TOPscript™ cDNA synthesis Kit (Enzynomics, Korea). After cDNA products were mixed with SYBR Premix Ex Taq II (TaKaRa, Japan), forward and reverse primers for RFP gene, or β-actin, DNA was amplified by using a StepOnePlus real-time PCR system (Applied Biosystems, US). Using β-actin as the house keeping gene, the 2^−ΔΔCt^ method was used to analyze the relative changes in the level of RFP mRNA from qRT-PCR experiments^45^.

For western blotting analysis, we lysed the cells with RIPA buffer containing cocktail protease inhibitors, and subsequently centrifuged the lysates. Equal amounts of total proteins were resolved by SDS-PAGE, and then transferred to PVDF membranes. Next, the membrane was blocked in 5% skim milk in TBST (10 mM Tris-HCl, pH8.0, 150 mM NaCl, 0.05% Tween-20) for 1 h, and further incubated with the primary antibodies specific for RFP or β-actin for 4 h at 4 °C. After washed three times with TBST, the membranes were incubated with HRP-conjugated secondary antibodies according to an enhanced chemiluminescence system, eventually leading to development of the blots. The specified bands could be visualized by using Image J software and EZ-Capture MG.

### *In vivo* biodistribution studies of auto_shRFP@LS

B16F10 melanoma cells (1 × 10^7^ cells/mouse) were subcutaneously inoculated to the dorsal side of female BALB/c nude mice (five-week-old), and subsequently grown until the tumor volume became approximately 90 mm^3^. Cy5.5-auto_shRFP@LS lipoplexes (2 mg/kg, 200 μL per mouse; three mice) or equivalent amounts of free Cy5.5-siRFP were intravenously administered into tail vein of B16F10-bearing xenograft mice. Mice were sacrificed 1 day post-injection to excise organs and tumors, and then the *ex vivo* images were analyzed using IVIS Spectrum (Caliper Life Science Inc., US) and IVIS Living Imaging Software. All the animal care and experimental procedures were carried out under a protocol approved by the KIST (Korea Institute of Science and Technology) Institutional Animal Care and Use Committee (Ref. No.: KIST2018-039). All animal experiments were performed in accordance with the recommendations for the proper use and care of the specific pathogen-free housing facility at KIST.

### *In vivo* gene silencing studies on xenograft mice

To monitor the *in vivo* RFP gene silencing efficiency of auto_shRFP@LS, the cultured B16F10/RFP cells (1 × 10^7^ cells/mouse) were subcutaneously inoculated into the dorsal sides of five-week-old female BALB/c nude mice. The RFP fluorescence signals measured from the tumor tissues showed that up to three weeks, their fluorescence intensities proportionally increased as the number of tumor cells increased. All the RFP emission signals from tumor tissues were normalized to photons per second per centimeter squared per steradian (p/s/cm2/sr), while all the illumination setting conditions including exposure time (10 seconds for all fluorescence images), filter, and lamp voltage were fixed identical throughout the animal imaging experiments. When the growth of RFP-expressing tumors reached 5–7 mm in diameter a week after tumor cell inoculation, the fluorescent signals at tumor sites were noninvasively measurable by using IVIS Spectrum and IVIS Living Imaging Software. Auto_shRFP@LS complexes were intravenously administered into tail-vein only once (54 μg per injection), and subsequently the RFP fluorescence intensities at tumors were measured at the specified time points. Similarly, scrambled shRFP-containing auto_shRFP@LS (54 μg per injection), PBS, or siRFP@LS (54 μg per injection) were intravenously injected only once, and finally the RFP fluorescence intensities from tumors were compared (n = 3 for each group). Mice were euthanized 6 days post-injection to obtain their *ex vivo* fluorescence images as well as to perform the additional *ex vivo* assays. For an immunohistochemical staining assay, paraffin-embedded tumor sections with 6 μm thickness were stained using anti-RFP antibodies and Histostain-Plus kit (Invitrogen, US). For quantitative determination of RFP mRNA present within the excised tumors, tumor tissues were homogenized and lysed. The RNA extraction and qRT-PCR reactions were performed according to the procedures similar to those described above. The relative amounts of RFP mRNA were normalized with respect to those of β-actin mRNA.

## Supplementary information

Supplementary Information
