# Supplementary material for: A T7 autogene-based hybrid mRNA/DNA system for long-term shRNA expression in cytoplasm without inefficient nuclear entry
Source: Sci Rep. 2019 Feb 28;9:2993. doi: 10.1038/s41598-019-39407-8 (PMC6395690; doi:10.1038/s41598-019-39407-8)
Supplement: Supplementary file 1 — Supplementary Information [file 41598_2019_39407_MOESM1_ESM.docx]

Supplementary Information

**A T7 autogene-based hybrid mRNA/DNA system for long-term shRNA expression in cytoplasm without inefficient nuclear entry**

Seo Young Kwak, Hee Dong Han and Hyung Jun Ahn


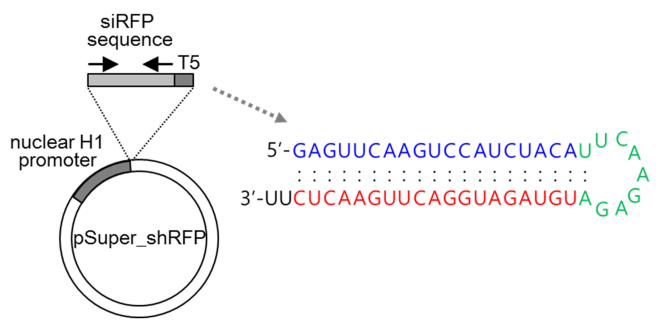


**Figure S1. Schematic illustration of RNA hairpin construct produced by nuclear pH1-driven shRNA expression.** pSuper_shRFP plasmid was constructed to express shRFP hairpin in the nuclear compartment, in a way that siRFP target sequence was located downstream of RNA polymerase III H1 promoter and upstream of termination site. At the termination site, which is composed of five T nucleotides, the nuclear transcript is cleaved after the second uridine, resulting in a short hairpin RNA with 19-bp stem region, 9-nt loop region and two 3′ overhanging U nucleotides.

**
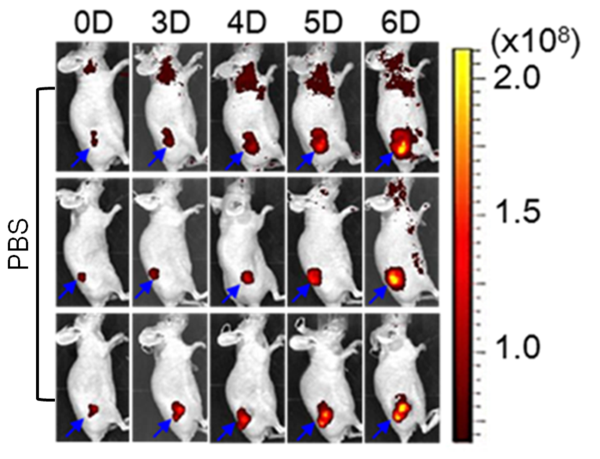
**

**Figure S2. Scrambled auto_shRFP@LS injected on B16F10/RFP tumor-bearing mice.** A matched control of Figure 6A corresponding to PBS-injected mice is shown here. The PBS-injected mice showed the proportional increase of RFP fluorescence intensity to the tumor growth on noninvasive fluorescence images at the indicated time points.


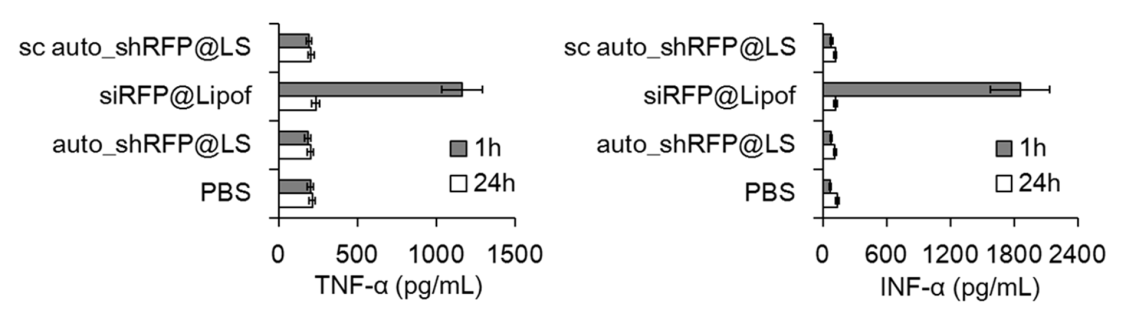


**Figure S3. Serum immunogenicity studies of auto_shRFP@LS in C57BL/6J mice.** Female C57BL/6J mice were intravenously injected with auto_shRFP@LS (54 μg per injection), scrambled auto_shRFP@LS (54 μg per injection), PBS (5 μg per injection) or siRFP@Lipofectamine (54 μg per injection), and then proinflammatory cytokine induction including TNF-α and INF-α was examined on the blood samples 1 h and 24 h post-injection. The results represent the mean ± s.d. (n = 3 mice per group).


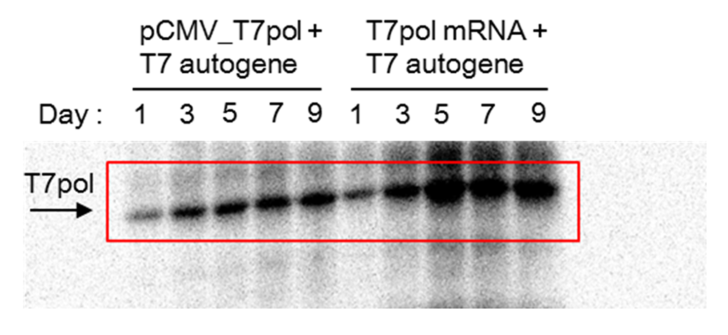


**Figure S4. The expression levels of T7pol protein in B16F10/RFP cells transfected by lipoplexes containing T7 autogene plasmids with either T7pol mRNAs or pCMV_T7pol plasmids.** The representative full blot image of Figure 3A is shown here.


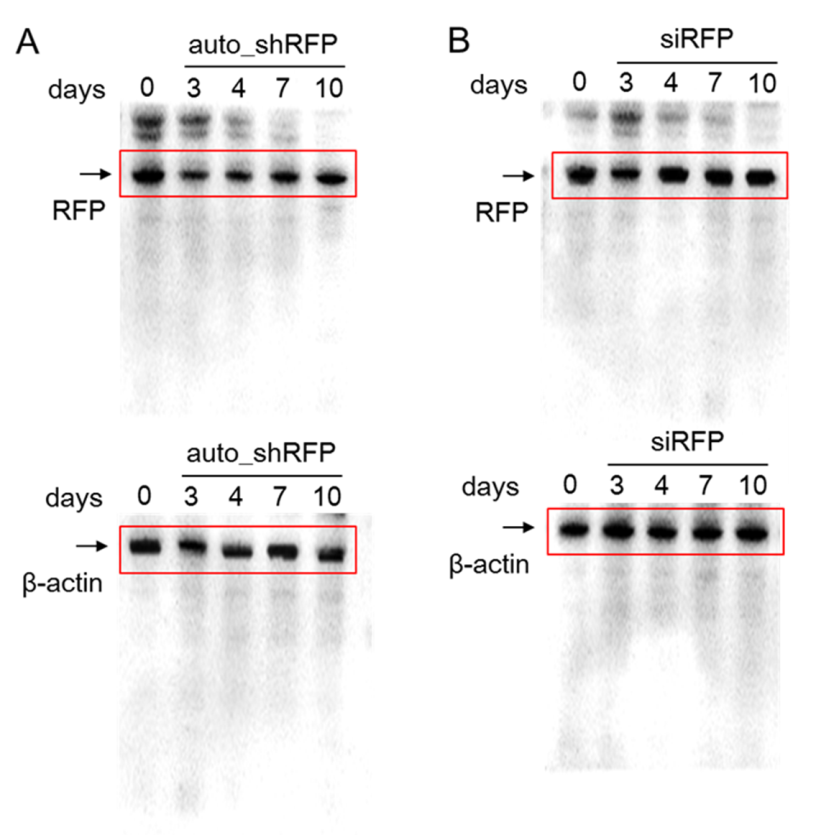


**Figure S5. The expression levels of RFP fluorescence protein and β-actin in auto_shRFP@LS-transfected B16F10/RFP cells.** The representative full blot images of Figure 4B are shown here.
